# Genetic Characterisation of *Plasmodium falciparum* Isolates with Deletion of the *pfhrp2* and/or *pfhrp3* Genes in Colombia: The Amazon Region, a Challenge for Malaria Diagnosis and Control

**DOI:** 10.1371/journal.pone.0163137

**Published:** 2016-09-16

**Authors:** Erika Jimena Dorado, Sheila Akinyi Okoth, Lidia Madeline Montenegro, Gustavo Diaz, John W. Barnwell, Venkatachalam Udhayakumar, Claribel Murillo Solano

**Affiliations:** 1 Malaria Research Group, Centro Internacional de Entrenamiento e Investigaciones Medicas (CIDEIM), Cali, Valle, Colombia; 2 Malaria Branch, Division of Parasitic Diseases and Malaria, Center for Global Health, Centers for Disease Control and Prevention (CDC), Atlanta, Georgia, United States of America; 3 Atlanta Research and Education Foundation, Decatur, Georgia, United States of America; Université Pierre et Marie Curie, FRANCE

## Abstract

Most *Plasmodium falciparum*-detecting rapid diagnostic tests (RDTs) target histidine-rich protein 2 (PfHRP2). However, *P*. *falciparum* isolates with deletion of the *pfhrp2* gene and its homolog gene, *pfhrp3*, have been detected. We carried out an extensive investigation on 365 *P*. *falciparum* dried blood samples collected from seven *P*. *falciparum* endemic sites in Colombia between 2003 and 2012 to genetically characterise and geographically map *pfhrp2-* and/or *pfhrp3*-negative *P*. *falciparum* parasites in the country. We found a high proportion of *pfhrp2*-negative parasites only in Amazonas (15/39; 38.5%), and these parasites were also *pfhrp3*-negative. These parasites were collected between 2008 and 2009 in Amazonas, while *pfhrp3*-negative parasites (157/365, 43%) were found in all the sites and from each of the sample collection years evaluated (2003 to 2012). We also found that all *pfhrp2*- and/or *pfhrp3*-negative parasites were also negative for one or both flanking genes. Six sub-population clusters were established with 93.3% (14/15) of the *pfhrp2*-negative parasites grouped in the same cluster and sharing the same haplotype. This haplotype corresponded with the genetic lineage B_V1_, a multidrug resistant strain that caused two outbreaks reported in Peru between 2010 and 2013. We found this B_V1_ lineage in the Colombian Amazon as early as 2006. Two new clonal lineages were identified in these parasites from Colombia: the genetic lineages E_V1_ and F. PfHRP2 sequence analysis revealed high genetic diversity at the amino acid level, with 17 unique sequences identified among 53 PfHRP2 sequences analysed. The use of PfHRP2-based RDTs is not recommended in Amazonas because of the high proportion of parasites with *pfhrp2* deletion (38.5%), and implementation of new strategies for malaria diagnosis and control in Amazonas must be prioritised. Moreover, studies to monitor and genetically characterise *pfhrp2*-negative *P*. *falciparum* parasites in the Americas are warranted, given the extensive human migration occurring in the region.

## Introduction

In the region of the Americas, about 121 million people are estimated to be at risk for malaria (with 20 million considered to be at high risk). In 2013, Brazil, Venezuela and Colombia accounted for 77% of cases in the Americas [1]. Colombia borders these countries to the East and shares the Amazon region with them along with Peru and Ecuador to the South. In Colombia, the major *Plasmodium* species are *P*. *falciparum* (50% of the malaria cases) and *P*. *vivax* (50% of cases) [1]. *P*. *falciparum* causes the most severe form of malaria. Most of the malaria cases in Colombia occur in remote areas with limited access. *P*. *falciparum* cases are more prevalent in the Colombian Pacific coast (which includes the Departments of Nariño, Choco, Cauca and Valle) and the Departments of Antioquia and Amazonas [2].

Light microscopy remains the gold standard for the field diagnosis of malaria. However, microscopic diagnosis of malaria is limited in some malaria endemic regions, especially in remote areas, and also in non-endemic countries. Rapid diagnostic tests (RDTs) are a useful alternative tool for malaria diagnosis. Malaria RDTs use antibody binding proteins that are produced by malaria parasites and can be detected in a small amount of infected blood. The targeted antigens used for species-specific detection are histidine-rich protein 2 (HRP2) or lactate dehydrogenase (LDH) enzymes of *P*. *falciparum* and *P*. *vivax*, respectively. For genus-specific detection, pan-species epitopes in the pLDH or aldolase enzymes are targeted [3, 4]. The most common antigen targeted by malaria RDTs is PfHRP2, representing more than 80% of malaria RDT products available on the market [3, 5]. PfHRP2 is a *P*. *falciparum* specific protein that is encoded by the subtelomeric gene *pfhrp2* located on chromosome 8. This protein contains a histidine-alanine-rich repeat region and is released as a soluble protein in the blood of infected individuals [6, 7]. In addition, it has been reported that *P*. *falciparum* Histidine-Rich Protein 3 (PfHRP3) shares many structural similarities with PfHRP2 and the former can sometimes be detected by some PfHRP2-detecting RDTs [8, 9].

One of the major potential limitations of using HRP2 based RDTs in the field is the presence of *P*. *falciparum* isolates with deletion of the *pfhrp2* and its homolog gene, *pfhrp3*, in the Americas [10–15]. The same phenomenon, albeit to a lesser extent, has been reported in Mali [16], Senegal [17], and India [18]. For instance, a false-negative PfHRP2-based RDT result on a malaria patient who had travelled to Brazil initially led to inappropriate disease management; it was later determined that the infecting isolate was both *pfhrp2-* and *pfhrp3*-negative [10]. In the Peruvian Amazon, a study revealed that 41% of the 148 *P*. *falciparum* isolates tested showed deletion of the *pfhrp2* gene, 70% showed deletion of the *pfhrp3* gene and 21.6% showed deletion of both genes [11]. In contrast, in Suriname higher levels of *pfhrp2* gene deletion than *pfhrp3* gene deletion were reported [12]. The proportion of *P*. *falciparum* parasites lacking these genes was also investigated in French Guiana [13] and Honduras [14], but only the deletion of the *pfhrp3* gene was observed in those countries: 7.4% of the isolates examined in French Guiana (n = 221) and 44.1% of those tested in Honduras (n = 68). In addition, studies have shown that the deletions of *pfhrp2* and *pfhrp3* genes are usually accompanied by deletions of one or both of their respective flanking genes [11, 12, 15]. Previously, we carried out a pilot evaluation of 100 historic samples from Colombia collected between 1999 and 2009, to investigate the presence of *pfhrp2*- and *pfhrp3*- negative parasites in the country. The pilot study revealed that 18% and 52% of the samples showed deletion of the *pfhrp2* and *pfhrp3* genes, respectively [15]. Since this was a pilot study with its small sample size restricted to only some parts of Colombia, further studies are required to get a better understanding of the distribution of *pfhrp2* deleted parasites in Colombia.

Extensive diversity in PfHRP2 amino acid sequences has been observed, both between and within countries worldwide [8, 9, 19, 20]. It has been suggested that the diversity in PfHRP2 could affect the sensitivity of PfHRP2-based RDTs at low parasite densities [9]. Similarly, variations have been found in PfHRP2 expression and *pfhrp2* and *pfhrp3* transcription levels between *P*. *falciparum* strains [21]. In addition, the evaluation of 22 *P*. *falciparum* blood samples using PfHRP2-enzyme-linked immunosorbent assays (ELISA), showed a large variation of antigen concentration between the samples (samples diluted at the same parasite density of 200 parasites/μL of blood) and it was suggested that this variation could be a consequence of amino acid sequence variations in PfHRP2 [22].

In the present study, we carried out a molecular surveillance study to further characterise deletion patterns of *pfhrp2* and/or *pfhrp3* genes in Colombia by utilising 365 *P*. *falciparum* dried blood samples collected retrospectively and prospectively from seven *P*. *falciparum* endemic sites between 2003 and 2012. The main goal of this investigation was to genetically characterise and geographically map this parasite population in Colombia, in order to: (1) understand the geographical distribution of *pfhrp2/pfhrp3* deleted parasites in the country; (2) determine if *pfhrp2/pfhrp3* deleted parasites have expanded clonally from common founding populations and characterise genetic relationship between *pfhrp2*-negative parasites found in Colombia with that of Peruvian *pfhrp2* deleted parasites; and (3) determine the genetic diversity of PfHRP2 sequence at the amino acid level.

## Materials and Methods

### Ethics statement

This study, and the use of all the samples included in the present study, was approved by the Institutional Ethics Committee on Human Research of the Centro Internacional de Entrenamiento e Investigaciones Medicas (CIDEIM). All the patients were older than or equal to seven years of age and participated in the study voluntarily. An informed consent form was signed by each participant and, in the case of minors (<18 years old), by the parent or legal guardian. In this case an assent form was also signed by the minor. All patients received antimalarial treatment according to Colombia’s national guidelines for malaria, independent of their enrolment in the study. Investigators from the Centers for Disease Control and Prevention (CDC) were non-engaged participants in this study as they did not have any contact with human subjects or access to personal identifying information of study subjects.

### Study population and sample collection

**Fig 1:**
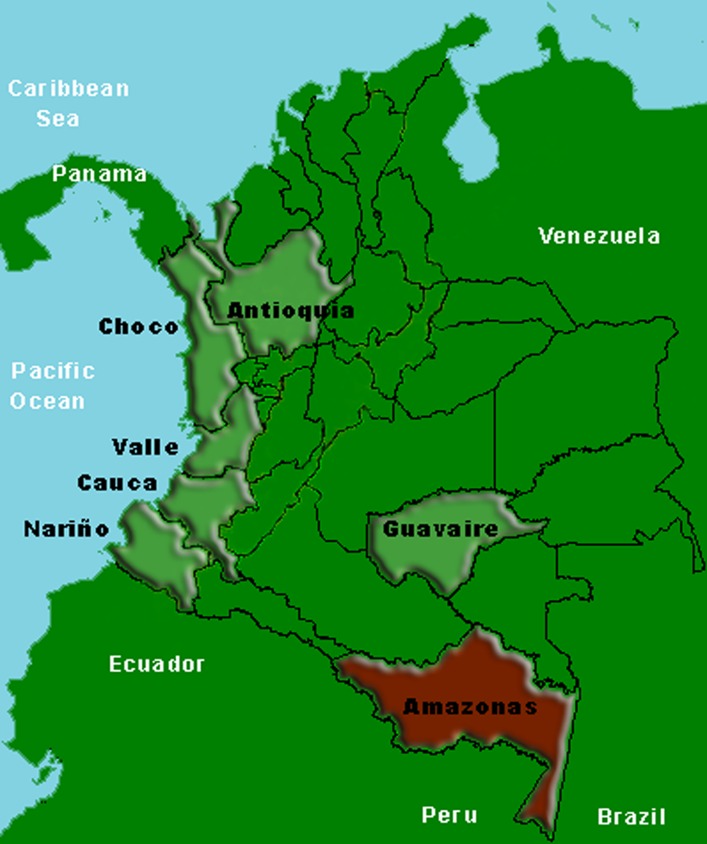
Sample collection sites in Colombia. On the map the sample collection sites are highlighted. The names in black indicate the seven Colombian Departments from which the isolates studied were collected: Antioquia (N = 42), Choco (N = 74), Valle (N = 27), Cauca (N = 44), Nariño (N = 122), Guaviare (N = 26) and Amazonas (N = 39). The study site highlighted in red, Amazonas, indicates the Department where *pfhrp2/pfhrp3* double negative isolates were found. Map source: DIVA-GIS, available in http://www.diva-gis.org/.

One hundred and fourteen dried blood spot samples collected between 2011 and 2012 from symptomatic patients diagnosed with *P*. *falciparum* malaria by microscopy were studied prospectively. These samples were collected in seven Departments, each of them with high numbers of *P*. *falciparum* malaria cases, and these Departments were distributed across several different Colombian regions: Antioquia in northern Colombia, Amazonas in southern Colombia, Guaviare in south-eastern Colombia, and Nariño, Choco, Cauca and Valle in the Colombian Pacific coast (the region with the highest number of *P*. *falciparum* malaria cases in the country [2]) (Fig 1). The patient enrolment process was carried out by trained personnel in health service centres or in the remote sites where the infections were reported. All patients met the following criteria: older than or equal to seven years of age and diagnosed with uncomplicated *P*. *falciparum* malaria by microscopy, with a parasitaemia between 500–100,000 parasites/μL. The blood samples were collected by finger prick or by venipuncture into ethylenediaminetetraacetic acid (EDTA) vacutainer tubes (Becton-Dickinson, USA), depending on the study site where the samples were collected. Thin and thick films on microscope slides and blood spots on Whatman no. 3 filter paper (Whatman^TM^, USA) were obtained for all the samples. In addition, 260 dried blood spot samples collected from patients diagnosed with *P*. *falciparum* malaria by microscopy during previous studies conducted by the malaria research group of CIDEIM, were evaluated. These samples were collected between 2003 and 2010 in the same Departments mentioned above.

### Malaria diagnosis by microscopy and RDT

Thick and thin blood films were obtained for all the prospective samples. Both the thick and thin films were stained with Giemsa (10% Giemsa for 10 minutes and 5% Giemsa for 20 minutes, respectively). The *P*. *falciparum* malaria diagnosis and parasitaemia were determined by microscopists in the study site, and later confirmed at CIDEIM. The parasitaemia was calculated by counting the number of parasites observed per 200 leukocytes, and assuming a total of 8,000 leukocytes/μL blood. These samples were also tested using two PfHRP2-based RDTs: SD Bioline Malaria Ag P.f/P.v (Standard Diagnostics, Inc., Korea) and CareStart™ Malaria HRP2/pLDH (Pf/PAN) combo (Access Bio, Inc., USA). These RDTs also detect pLDH: SD Bioline detects pLDH specifically for *P*. *vivax* and CareStart™ Malaria is pan-pLDH based. The RDTs were used according to manufacturer’s instructions.

### DNA extraction and confirmation of *P*. *falciparum* infections by PCR

Genomic DNA was extracted from dried blood spots for all the 374 samples studied using the QIAamp® DNA Micro kit (QIAGEN, USA) following the manufacturer's instructions. The 18S rRNA gene was amplified by a semi-nested multiplex-PCR described previously [23], to confirm *P*. *falciparum* infections and test the DNA quality of all 374 samples. Additionally, DNA quality was confirmed by amplification of the *P*. *falciparum* merozoite surface protein 1 (*pfmsp1*), *pfmsp2* and glutamate-rich protein *(pfglurp)* genes [24].

### Detection of *pfhrp2*, *pfhrp3* and their flanking genes

Once *P*. *falciparum* infection and DNA quality were confirmed, the presence of *pfhrp2* and *pfhrp3* in the isolates was determined by amplifying across exon 1 and 2 of both genes. Similarly, genes flanking *pfhrp2* (PF3D7_0831900 and PF3D7_0831700) and *pfhrp3* (PF3D7_1372100 and PF3D7_1372400) were PCR amplified to characterise the extent of the chromosomal deletions in the *pfhrp2*- and/or *pfhrp3*-negative parasites. The PCR conditions and primers were described previously [14]. The PCR products were separated by electrophoresis on a 1.5% agarose gel stained with ethidium bromide. The bands were then visualized under UV light using the Gel Doc^TM^ 2000 system (Bio-Rad Laboratories, Inc., USA) and analysed with Quantity One Software version 4.6.3 (Bio-Rad Laboratories, Inc., USA). The samples were reported as negative for the specific gene after two failed amplification attempts. Amplification was undertaken a third time if the second PCR result was discordant with the first. Any two concordant results were recorded as the final outcome.

Genomic DNA of three *P*. *falciparum* laboratory strains were included as controls in the analysis (3D7, Dd2 and HB3). Laboratory strain 3D7, was used as a positive control for both *pfhrp2* and *pfhrp3* amplification. Laboratory strain Dd2, which lacks *pfhrp2* and its flanking genes, was used as a negative control for *pfhrp2* amplification and as a positive control for *pfhrp3* amplification. Laboratory strain HB3, which lacks *pfhrp3* and its flanking genes, was used as a negative control for *pfhrp3* amplification and as a positive control for *pfhrp2* amplification.

### Microsatellite genotyping and cluster analysis

Microsatellite genotyping was carried out on *pfhrp2*- and/or *pfhrp3*-negative isolates. Seven neutral microsatellite markers distributed across six chromosomes were evaluated: C2M34 (chromosome 2), C3M69 (chr. 3), Polyα (chr. 4), TA1 (chr. 6), TA109 (chr. 6), 2490 (chr. 10) and PfPk2 (chr. 12). The neutral microsatellite loci and the amplification conditions were previously described [25–28]. Microsatellite alleles in each of the loci were amplified using fluorescently labelled primers and size differentiation was performed by capillary electrophoresis on an ABI 3130xl sequencer (Applied Biosystems, USA). The allele lengths were scored using GeneMapper® version 3.7 software (Applied Biosystems, USA), using the default settings for microsatellite analysis. Only samples with a minimum peak height of 100 relative fluorescent units and one allele at each locus were included in further analyses.

Bayesian STRUCTURE version 2.3.3 software [29] was used to determine the possible population structure among the isolates included. The STRUCTURE parameters used were as follows: an admixture model with correlated allele frequencies, a 10,000 burn-in period followed by 100,000 Markov Chain Monte Carlo (MCMC) repetitions for 20 iterations and testing for the most likely number of clusters (*K*) that best fit the data between 1 and10. Similar parameters were previously used for the population structure analysis of *P*. *falciparum* in Colombia [30], Peru [31] and Honduras [14]. The most likely value of *K* for this population was defined by the STRUCTURE Harvester programme implementing the Evanno method [32].

### PfHRP2 and PfHRP3 sequence polymorphisms

Exon 2 of *pfhrp2* and *pfhrp3* was sequenced and analysed in *pfhrp2*- and *pfhrp3*-positive isolates, following the method previously described by Baker *et al*. [9]. PCR products were sent to Macrogen Inc. (Korea) for sequencing and the nucleotide sequences obtained were translated to amino acid sequences. BioEdit software version 7.0.9.0 was used for amino acid sequence analysis [33]. The 3D7 amino acid sequence available in the PlasmoDB database (www.plasmodb.org) was used as reference. The type and number of repeats in the histidine-alanine-rich repeat region were determined according to the types of repeats found in PfHRP2 and PfHRP3 amino acid sequences [9].

## Results

Three hundred and seventy-four samples from symptomatic patients diagnosed with uncomplicated *P*. *falciparum* malaria by microscopy were evaluated. The samples were collected between 2003 and 2012 in seven *P*. *falciparum* malaria endemic Departments of Colombia. The mean age of the patients was 28.2 (SD = 14.6, median = 26, range = 7–66), 63.1% were male and 36.9% female.

### Microscopy and malaria RDTs

All of the 114 thick blood films obtained from the prospective samples were reported positive for *P*. *falciparum* by microscopy in the collection site and were confirmed at CIDEIM. Overall, the parasitaemias ranged between 500 and 73,760 parasites/μL, with a mean of 12,046 parasites/μL and median of 6,800 parasites/μL. Fifty of the 114 samples from five study sites (Amazonas, Antioquia, Cauca, Choco and Valle) were tested using CareStart™ Malaria and SD Bioline Malaria Ag P.f/P.v RDTs. Only samples with validated reading reports for both RDTs were included in this analysis. The remaining samples were tested using only one of the RDTs mentioned above or were tested with another type of RDT because of delays in the supply of the RDTs in the remote areas where the samples were collected.

CareStart™ Malaria RDT detected PfHRP2 in 49 of the 50 samples evaluated. The sample that was negative for PfHRP2 had 2,920 parasites/μL and was pan-pLDH positive. This sample was identified as *P*. *falciparum* by PCR, microscopy and SD Bioline Malaria RDT. SD Bioline Malaria RDT detected PfHRP2 in all of the 50 samples evaluated. Three of these 50 samples were reported as *P*. *falciparum/P vivax* mixed infections by SD Bioline Malaria RDT (both PfHRP2 and Pv-pLDH were detected). The three mixed infections were identified as *P*. *falciparum* infections by PCR and microscopy (Table 1).

**Table 1:** Samples with RDT results that differed from PCR results.

Study site	Sample ID	Parasitaemia (parasites/μL)	RDT result	
			SD Bioline	CareStart™
Choco	SL007	520	**P.f-HRP2 (+)/Pv-pLDH (+)**	P.f-HRP2 (+)/Pan-pLDH (+)
Choco	SL013	1000	**P.f-HRP2 (+)/Pv-pLDH (+)**	P.f-HRP2 (+)/Pan-pLDH (+)
Choco	SL015	780	**P.f-HRP2 (+)/Pv-pLDH (+)**	P.f-HRP2 (+)/Pan-pLDH (+)
Valle	VAL01	2920	P.f-HRP2 (+)/Pv-pLDH (-)	**P.f-HRP2 (-)/Pan-pLDH (+)**

The RDT results which differed from the PCR outcomes are highlighted in bold. The five samples were identified as *P*. *falciparum*-positive by both PCR and microscopy.

### Confirmation of *P*. *falciparum* infections by PCR

Three hundred and seventy of the 374 samples collected met the inclusion criteria, those inclusion criteria being positive amplification by PCR of 18S rRNA, *pfmsp1*, *pfmsp2* and *pfglurp* genes. Amplification of the 18S rRNA gene showed that five of the 370 samples were mixed *P*. *falciparum*/*P*. *vivax* infections. Therefore, 365 out of the 374 samples initially tested were included in the study. The 365 samples consisted of 112 prospective and 253 retrospective samples.

### Proportion of *P*. *falciparum* parasites with *pfhrp2* and/or *pfhrp3* gene deletions

*Pfhrp2*-negative isolates were not found within the prospective samples. Fifty one of the 112 (45.5%) prospective samples were *pfhrp3*-negative (Table 2). All of the *pfhrp3*-negative isolates were also negative for the downstream flanking gene PF3D7_1372400 and most of them (46/51; 90.2%) were also negative for the upstream flanking gene PF3D7_1372100.

**Table 2:** Prospective samples (collected between 2011 and 2012): Proportion of isolates with deletions of *pfhrp3* and its respective flanking genes, per site and total.

Department	N	PF3D7_1372100	*pfhrp3*	PF3D7_1372400
Amazonas	11	3	3 (27.3)	3
Antioquia	22	21	21 (95.5)	21
Cauca	11	1	2 (18.2)	2
Choco	28	8	8 (28.6)	8
Guaviare	9	7	8 (88.9)	8
Nariño	30	6	9 (30)	9
Valle	1	0	0	0
Total	112	46 (41.1)	51 (45.5)	51 (45.5)

The numbers in each box indicate the number of isolates with deletion of the respective gene, while the numbers in parenthesis indicate the proportions, as percentages, per site. The last row shows the total number of isolates with deletions of the respective genes, and the numbers in parenthesis indicate the number of deletions as a percentage of the total number of isolates.

Fifteen *pfhrp2*-negative isolates were found within the retrospective samples: 15 of the 28 (53.6%) samples collected from Amazonas (Fig 1, Table 3). These isolates were collected between 2008 and 2009, and were also *pfhrp3*-negative. These *pfhrp2/pfhrp3* double negative isolates were also negative for the *pfhrp2* upstream flanking gene (PF3D7_0831900) and *pfhrp3* downstream flanking gene (PF3D7_1372400). One hundred and six of the 253 (41.9%) retrospective samples evaluated were *pfhrp3*-negative. Most of them (101/106; 95.3%) were also negative for the downstream flanking gene PF3D7_1372400 and 52.8% (56/106) were negative for the upstream flanking gene PF3D7_1372100.

**Table 3:** Retrospective samples (collected between 2003 and 2010): portion of isolates with deletions of *pfhrp2*, *pfhrp3* and their respective flanking genes, per site and total.

Department	Year	N	PF3D7_0831900	*pfhrp2*	PF3D7_0831700	PF3D7_1372100	*pfhrp3*	PF3D7_1372400
Amazonas	2008–2010	28	15	15 (53.6)	0	5	20 (71.4)	20
Antioquia	2008–2010	19	0	0	0	19	19 (100)	17
Cauca	2003	33	0	0	0	4	4 (12.1)	2
Choco	2008–2010	42	0	0	0	5	5 (11.9)	4
Guaviare	2009–2010	15	0	0	0	9	15 (100)	15
Nariño	2008–2010	90	0	0	0	14	37 (41.1)	37
Valle	2004–2006	26	0	0	0	0	6 (23.1)	6
Total	2003–2010	253	15 (5.9)	15 (5.9)	0	56 (22.1)	106 (41.9)	101 (39.9)

The numbers in each box indicate the number of isolates with deletion of the respective gene, while the numbers in parenthesis indicate the proportions, as percentages, per site. The last row shows the total number of isolates with deletions of the respective genes, and the numbers in parenthesis indicate the number of deletions as a percentage of the total number of isolates.

### Microsatellite genotyping and cluster analysis

Microsatellite profiles were determined for 161 *pfhrp2*-negative and/or *pfhrp3*-negative isolates. One hundred and thirty two of the 161 samples initially analysed were identified as monomorphic based on the microsatellite data, and the allele sizes were scored for most of the loci evaluated. The geographic distribution (by Department) of the 132 isolates was as follows: 40 isolates were collected in Nariño, 39 from Antioquia, 23 from Amazonas, 15 from Guaviare, seven from Choco, five from Cauca and three from Valle. A complete microsatellite profile was obtained for 95.5% of the samples (126/132 samples). The remaining six samples had missing allele size information in one or two loci. The range of alleles per locus was between two and 12 alleles. The most polymorphic locus in this sample set was Polyα with 12 alleles, while the least polymorphic was TA109 with two alleles.

**Fig 2:**
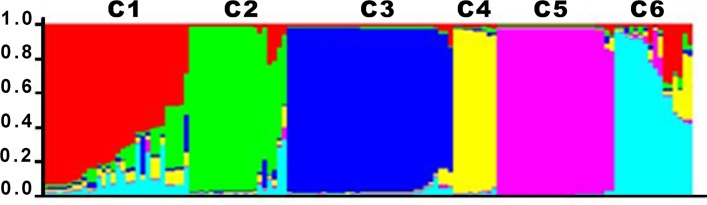
Clusters (C1—C6) defined by STRUCTURE v2.3.3. for the population of *pfhrp2-* and/or *pfhrp3*-negative parasites in Colombia (N = 132). Each isolate (*x*-axis) is represented by a single vertical bar, and this bar is broken down into various coloured segments whose length varies according to the membership fraction (*y-* axis) of each of the six inferred clusters (K = 6). Cluster 1 (red) contained 22.7% of the total isolates evaluated (30 isolates). These were mainly samples from the Pacific coast and northern Colombia. Cluster 2 (green) contained 14.4% of the samples evaluated (19 isolates). Ninety-five percent of the samples in Cluster 2 were from northern Colombia. Cluster 3 (blue) contained 25.8% of the samples evaluated (34 isolates), all of which were collected in in the Colombian Pacific coast. Cluster 4 (yellow) was the smallest cluster with 6.8% of the samples (9 isolates). These originated from Guaviare, Nariño and Choco. Cluster 5 (pink) contained 18.2% of the samples (24 isolates) and they were collected in southern and south-eastern Colombia. This cluster contained the *pfhrp2*/*pfhrp3* double negative samples. Cluster 6 (aqua) included 12.1% of the samples evaluated (16 isolates) and they originated from all the sites studied except Nariño.

In total, 51 haplotypes were identified and were distributed as follows: Nariño (n = 38; 10 haplotypes), Antioquia (n = 36; 19), Amazonas (n = 22; 7), Guaviare (n = 15; 9), Choco (n = 7; 5), Cauca (n = 5; 4). Guaviare shared two haplotypes with other sites (one each with Choco and Nariño) while Antioquia and Nariño shared one haplotype. All haplotypes in Amazonas were unique to that site. STRUCTURE analysis predicted the presence of at least six clusters among the 132 *pfhrp2*-negative and/or *pfhrp3*-negative isolates (Fig 2). All but one of the 15 *pfhrp2*-negative isolates were grouped into the same cluster (Cluster 5 –pink) and shared the same haplotype, corresponding to the genetic lineage B_V1_ previously observed in Peru (Table 4) [34, 35]. These 14 samples were collected in Amazonas between 2008 and 2009. Cluster 5 contained 18.2% (24/132 isolates) of the total isolates evaluated, these were collected in southern and south-eastern Colombia (Amazonas and Guaviare).

**Table 4:** Microsatellite profiles identified for *P*. *falciparum* isolates lacking *pfhrp2* and new *P*. *falciparum* genetic lineages identified in Colombia.

Location	*pfhrp2*	Genetic lineage	TA1 (Ch5)	Polyα (Ch4)	PfPK2 (Ch7)	TA109 (Ch6)	2490 (Ch10)	C2M34 (Ch2)	C3M69 (Ch3)
Peru (1998–2001) ^a^^,^^b^									
	Neg	A	169	172	166	164	84	240	132
	Neg	B	172	183	172	164	84	226	149
	Neg	C	178	164	163	160	80	246	136
	Neg	D	178	161	175	160	80	233	122
	Pos	E	172	148	175	160	74	226	138
Peru (2010–2012) ^c^									
	Neg	B_V1_	169/172	183	169/172	164	84	232	134
Amazonas (2008–2009, n = 14; 2009, n = 1) ^d^									
	Neg	B_V1_	172	183	172	164	84	232	134
	Neg	-	181	183	166	164	80	240	134
Amazonas (2006, 2007, n = 14) ^e^									
	Neg	B_V1_	172	183	172	164	84	232	134
		-	174	148	164	162	74	226	124
Cordoba (2010, n = 2) ^e^									
	Neg	-	172	158	175	162	80	236	124
Nariño (2010, n = 1) ^e^									
	Neg	-	172	179	175	160	80	226	140
Valle (2010, n = 1) ^e^									
	Neg	-	172	154	163	162	80	226	124
Antioquia (2012, n = 16) ^f^									
	Pos	E_V1_	172	148	175	160	74/75	236/226	124/140
Nariño (2008–2011, n = 26), Valle (2005–2006, n = 2), Cauca (2012, n = 2) ^f^									
	Pos	F	172	180	169/175	160	80	224/226	140

Ch = chromosome. (-) = microsatellite profile not defined as a genetic lineage.

^a,b^ Genetic lineages for *P*. *falciparum* parasites found in Peru [28].

^c^ Genetic lineage for *pfhrp2*-negative parasites found in Peru [34].

^d^ Genetic lineage for *pfhrp2*-negative parasites found in the present study.

^e^ Microsatellite profiles for *pfhrp2*-negative parasites found in our pilot study [15].

^f^ New genetic lineages for *P*. *falciparum* parasites found in the present study.

The largest cluster (Cluster 3 –blue) contained 25.8% (34/132) of the isolates evaluated (Fig 2). All the samples within this cluster were collected in the Colombian Pacific coast: 31 samples were from Nariño, two from Valle and one from Cauca. Eighty-five percent (29/34) of these isolates shared the same genetic lineage, and have been designated here as lineage F because, to our knowledge, this parasite haplotype has not been defined previously in *P*. *falciparum* parasites in South America (Table 4). Based on our findings, lineage F parasites were circulating in Colombia between 2005 and 2012.

The second largest cluster (Cluster 1 –red) contained 22.7% (30/132) of the total isolates evaluated, and consisted mainly of parasites from the Pacific coast and northern Colombia (Fig 2). Fifty-three percent of Cluster 1 specimens were from Antioquia, while the rest were collected in Nariño and Choco. Cluster 2 (green) contained 14.4% (19/132) of the samples evaluated (Fig 2). All but one of the samples from Cluster 2 were from Antioquia (northern Colombia). The remaining sample was collected in the Colombian Pacific coastal site in Cauca. Based on their microsatellite profile, 16 samples from Antioquia (12.1% of the total number of isolates analysed) collected in 2012 showed a variant of the clonal lineage E which was previously described for Peruvian isolates [34]. We have therefore named this variant lineage E variant-1 (E_V1_) because the alleles at loci 2490, C2M34 and C3M69 differ from those of E clonal lineage parasites (Table 4).

Cluster 6 (aqua) included 12.1% (16/132) of the isolates evaluated (Fig 2). These were collected in all the sites studied except Nariño. This cluster also included one of the *pfhrp2*-negative specimens from Amazonas that displayed a microsatellite profile that was different from that of the B_V1_ lineage that was predominant in that Department. Cluster 4 (yellow) was the smallest cluster with 6.8% (9/132) of the total isolates and included samples from Guaviare, Nariño and Choco. Mixed ancestry was observed between Clusters 1 and 2, with both clusters containing specimens from Antioquia Department (northern Colombia) (Fig 2). The predominant genetic lineages in this study were F (22% of the isolates), E_V1_ (12,1%), and B_V1_ (10%) (Table 4).

### PfHRP2 sequence variation

Fifty three nucleotide sequences with high quality sequencing data were analysed out of a total of 76 *pfhrp2* exon 2 sequences initially obtained. Overall, PfHRP2 sequence variation was evaluated in seven study sites: Nariño (15/22 sequences), Amazonas (10/10), Choco (9/14), Guaviare (8/10), Antioquia (6/10), Valle (3/5), and Cauca (2/5). The nucleotide sequence lengths ranged between 732 and 819 bp. The protein sequence lengths ranged between 244 and 273 amino acids. The most common amino acids in the PfHRP2 sequences were His (39.71–41.26% sequence composition), Ala (40.81–44.49%) and Asp (10.51–11.89%). Variation in the number and order of the repeat types was observed. All PfHRP2 sequences (100%) started with one to six type 1 repeats (AHHAHHVAD) and ended with one type 12 repeat (AHHAAAHHEAATH) (Table 5). The central part of the sequences was composed of types 2–8 and 10 repeats; however the order and number varied between sequences. Types 1, 2, 6, 7 and 12 repeats were present in all PfHRP2 sequences. The most common repeat types were type 2 (7 to 14 repeats per sequence) and type 7 (5 to 11 repeats per sequence). The type 9, 11 and 14 repeats were not observed in any sequence and the type 13 repeat was only observed twice, both in sequences of isolates that originated in Amazonas (Table 5).

**Table 5:** Number and type of repeats found in the PfHRP2 amino acid sequences.

Type of repeat	Amazonas (n = 10)	Antioquia (n = 6)	Cauca (n = 2)	Choco (n = 9)	Guaviare (n = 8)	Nariño (n = 15)	Valle (n = 3)
**1—AHHAHHVAD**	2–6	4–6	2	1–6	2–6	1–5	4
**2—AHHAHHAAD**	9–13	9–14	12	7–14	9–14	12–14	12
**3—AHHAHHAAY**	1–2	0–1	2	0–2	0–2	0–2	1
**4—AHH**	0–1	0–2	1	0–4	0–2	0–2	0
**5—AHHAHHASD**	0–2	0–1	1	0–2	0–2	0–1	1
**6—AHHATD**	2–5	2–5	5	3–7	2–7	3–5	4
**7—AHHAAD**	6–11	5–7	7	5–8	5–7	5–8	6
**8—AHHAAY**	0–1	1	1	0–2	0–1	0–2	1
**9—AAY**	0	0	0	0	0	0	0
**10—AHHAAAHHATD**	0–2	2–4	1	1–2	1–2	0–2	1
**11—AHN**	0	0	0	0	0	0	0
**12—AHHAAAHHEAATH**	1	1	1	1	1	1	1
**13—AHHASD**	0–1	0	0	0	0	0	0
**14—AHHAHHATD**	0	0	0	0	0	0	0

The numbers in each cell in the table indicate the range of the number of times that each type of repeat was present in each site evaluated.

**Fig 3:**
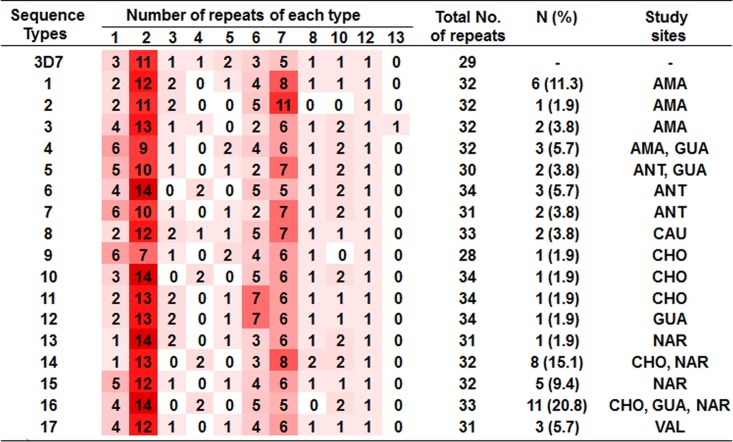
PfHRP2 sequence types identified in Colombia. Fifty three PfHRP2 sequences were evaluated and 17 unique sequences were identified. The heatmap shows the frequencies of the repeats for each type of PfHRP2 sequence identified. Study sites where the respective PfHRP2 sequence type (from 1 to 17) was found: AMA = Amazonas, GUA = Guaviare, ANT = Antioquia, CHO = Choco, NAR = Nariño, VAL = Valle. N = number of isolates that showed the PfHRP2 sequence type and (%) = percentage of the PfHRP2 sequence type in the population studied.

PfHRP2 sequence variation was observed within and among the study sites. 17 unique PfHRP2 sequences were identified among the 53 *pfhrp2* exon 2 sequences analysed (GenBank accession numbers KU723607- KU723623). The type and number of repeats for these 17 types of PfHRP2 sequences are shown in Fig 3. Among the nine sequences obtained from Choco isolates, five different sequence types were identified (sequence types 9, 10, 11, 14 and 16). Three different sequence types were identified among the six sequences from Antioquia samples (sequence types 5, 6 and7). Four different sequence types were identified among the eight sequences from Guaviare samples (sequence types 4, 5, 12 and 16). Four different sequence types were identified among the ten sequences from Amazonas samples (sequence types 1 to 4) and also among the 15 sequences obtained from Nariño samples (sequence types 13 to 16). One single type of PfHRP2 sequence (type 17) was identified in three Valle samples, and one single type of PfHRP2 sequence (type 8) was also identified in two Cauca samples (Fig 3).

### PfHRP3 sequence variation

Our exploratory study of PfHRP3 sequence variation was evaluated in six *pfhrp3*-positive samples from Amazonas (southern Colombia) and nine samples from Choco (Colombian pacific coast). The nucleotide sequences obtained ranged from 360 to 510 bp and the amino acid sequences lengths ranged from 170 to 210 aa. The most common amino acids in the PfHRP3 sequences were His (33.92–34.31% sequence composition), Ala (32.94–35.24%), Asn (10.59–11.11%) and Asp (9.05–10%). All the sequences showed seven out of the eight repeat types previously defined [9]. Repeat types 1, 4, 7, 15, 16, 17 and 18 were present, while repeat type 2 was absent. In total 4 PfHRP3 unique sequences were identified (GenBank accession numbers KC899082, KC899084, KC899085, KU723606). All the isolates from Amazonas showed the same sequence type, while three sequence types were observed in isolates from Choco. The variation among these sequences was defined by the number of repeats of type 16 and 17. All the sequences followed the same pattern: starting with one type 1 repeat, followed by one type 15 repeat (AHHAHHAAN), between 12 and 17 repeats of type 16 (AHHAAN), one type 7 repeat (AHHAAD), a non-repetitive sequence of 33 amino acids (the same amino acid sequence in all isolates), one type 17 repeat (AHHDG), one type 18 repeat (AHHDD), one type 17 repeat, one type 18 repeat, between 3 and 6 repeats of type 17 and all the sequences ended with the type 4 repeat (AHH).

## Discussion

Our extensive investigation of *P*. *falciparum* parasites lacking *pfhrp2* and/or *pfhrp3* genes in Colombia revealed a high prevalence of *pfhrp2*-negative parasites in Amazonas Department (38.5%, 15/39), southern Colombia (Fig 1). These parasites were also *pfhrp3*-negative. We also found that these isolates showed deletion of the *pfhrp2* upstream flanking gene, PF3D7_0831900, the same as *pfhrp2*-negative parasites reported in Peru [11]. Greater occurrence of *pfhrp3*-negative parasites (43%, 157/365) was found in isolates from the Colombian study sites investigated. For instance, in Antioquia and Guaviare Departments more than 95% of the samples evaluated showed deletion of the *pfhrp3* gene. These results are comparable to those found in Peru [11], but contrast with the results obtained in Suriname where higher levels of *pfhrp2*-negative than *pfhrp3*-negative parasites were found [12]. While *pfhrp2*-negative parasites were only observed within the retrospective samples collected in Amazonas between 2008 and 2009, *pfhrp3*-negative parasites were observed in all the sites studied and over all the years studied, from 2003 to 2012 (Tables 2 and 3). Although in our pilot study two *pfhrp2*-negative samples isolates were reported found in the Pacific coastal region of Colombia [15], this extensive investigation has provided additional and current evidence that the prevalence of this genetic deletion is rare in this region.

According to the epidemiological data obtained from patients with *pfhrp2*-negative parasites included in the present study, two samples collected in 2009 came from patients who reported to have travelled to Brazil prior to the infection. This suggests that as a consequence of the high migration of people between the countries bordering Amazonas, *pfhrp2*-negative parasites may be found in Brazil and Venezuela. The migration of people between the countries bordering Amazonas is known, and it is likely that the increased mining in the wider Amazon region could be a significant factor contributing to the importation of malaria parasites. As a consequence of the excavations made by miners, large areas fill with rainwater and contribute massively to mosquito breeding [36]. This may contribute to an increase in the number of malaria cases in the region, as well as the risk of malaria outbreaks.

Cluster analysis grouped the 132 *pfhrp2*-negative and/or *pfhrp3*-negative isolates evaluated into six clusters (K = 6). The population of *pfhrp2*-negative parasites, except one isolate, belonged to the same cluster (cluster 5—pink) (Fig 2). We compared the microsatellite profile from these samples with those obtained in our pilot study [15], and we found the same haplotype in *pfhrp2*-negative samples collected in Amazonas between 2006 and 2007 (samples evaluated in the pilot study). This parasite lineage, named B-variant 1 (B_V1_), is related to the clonal lineage B, the latter having previously been defined in specimens collected in the eastern Peruvian Amazon between 1999 and 2000 (Table 3) [34]. This B_V1_ lineage was previously observed in *pfhrp2*-negative parasites collected in Peru between 2010 and 2012, and it was also reported as a strain with multiple mutations in the *pfcrt*, *pfdhfr*, *pfdhps*, and *pfmdr1* genes, associated with chloroquine and sulfadoxine-pyrimethamine resistance [33]. Parasites with this shared haplotype were responsible for two outbreaks of *P*. *falciparum* malaria in Peru; one outbreak occurred in the northern coastal region of Peru between 2010 and 2012 [33] while the second occurred in Cusco Department, south-eastern Peru, in 2013 [34]. Therefore, our results show evidence for the presence of B_V1_ lineage parasites in the Colombian Amazon as early as 2006 (Table 4). Therefore, we could hypothesise that the B_V1_ lineage originated in some part of the Amazon region and human migration could have contributed to the widespread presence of this clonal lineage in different parts of Peru and in the Colombian Amazon.

In the present study, two new clonal lineages were defined. Firstly, the genetic lineage E_V1_, a variant of the clonal lineage E previously described in Peruvian isolates [28]. The parasites that belonged to this lineage were collected in 2012 in northern Colombia and the Colombian Pacific coast. Secondly, the genetic lineage F was found in the Colombian Pacific coast between 2005 and 2012 (Table 3). Further studies involving parasite isolates from the countries bordering Colombia will help to understand the population history of *P*. *falciparum* parasites in this region, and will assist malaria outbreak investigation and the study of imported malaria cases. It would be advisable to continue with the characterisation of *P*. *falciparum* clonal lineages in Colombia and define their antimalarial drug resistance profile for the molecular surveillance of *P*. *falciparum* in the country. This knowledge would contribute to the implementation of new strategies for malaria control, future vaccine performance studies and the monitoring of malaria cases in this new era of elimination and eradication of malaria.

There was a high PfHRP2 sequence diversity within the *pfhrp2*-positive parasites evaluated. Seventeen unique PfHRP2 sequences were identified within the 53 PfHRP2 sequences analysed (Fig 3). The most common repeats in the PfHRP2 sequences were types 2 and 7, while type 13 was only found in one sample from Amazonas. This repeat type 13 has only been observed in a sample from Peru, which was analysed in a similar study conducted worldwide [9]. This supports the fact that *P*. *falciparum* parasites in the Colombian Amazon are genetically similar to those found in Peru. Additionally, our exploratory PfHRP3 sequence analysis showed for the first time the composition of PfHRP3 sequences from Colombian isolates (given the sample size of six isolates, this would need to be re-confirmed by further studies). Lower sequence variation was observed for the PfHRP3 sequences when compared to PfHRP2 sequences. Moreover, we found that PfHRP2 and PfHRP3 protein sequences shared two types of repeats, types 1 and 7. This confirms the fact that PfHRP3 shares many structural similarities with PfHRP2 and we could hypothesise that the shared repeats type 1 and 7 could explain the cross reactivity between the proteins and PfHRP3 being detected by PfHRP2-detecting RDTs.

We used PCR as the reference test and found discrepancies between RDT results and PCR (Table 1). One false-negative result for *P*. *falciparum* identification was reported by CareStart™ Malaria (only the pan-pLDH antigen was detected), and three mixed infections (P. f/P. v) were reported by SD Bioline Malaria RDT. These samples were all identified as *P*. *falciparum* infections by PCR and microscopy, and were *pfhrp2*-positive. The mixed infections (P. f/P. v) observed could be due to a reaction between the antibodies on the RDTs and rheumatoid factor in the blood of the patients or due to the presence of other diseases like hepatitis C, dengue or leishmaniasis, amongst others [37]. We also found that both RDTs effectively detected PfHRP2 in the *pfhrp3*-negative samples (19 out of the 50 prospective samples evaluated by RDT were *pfhrp3*-negative samples). Therefore, despite the high PfHRP2 variability in Colombia and deletion of the *pfhrp3* gene, the PfHRP2-detecting RDTs were not affected. Similarly, discrepancies between microscopy and PCR were observed. Five samples reported as *P*. *falciparum* infections were detected as mixed infections (P. f/P. v) by PCR. Molecular techniques are more sensitive compared to both microscopy and RDTs, however PCR is not a rapid tool and is more expensive and requires specialised personnel and equipment. Therefore, the development of new malaria diagnosis techniques adaptable for use in the field should be prioritized. Examples of these are molecular-based isothermal tests like loop-mediated isothermal amplification (LAMP), which has high sensitivity and specificity like PCR. LAMP is a faster rapid diagnostic tool when compared to PCR, and has no requirement for expensive thermal cyclers, [38, 39]. Additionally, this method is suitable for malaria diagnosis from dried blood spots, making it more feasible to undertake studies in endemic countries where obtaining blood samples in the field is a difficult task due to the poor field conditions. Because of the challenging field conditions in Colombia, a PfHRP2-ELISA was not undertaken to confirm the absence of the protein in the *pfhrp2*-negatives samples found in the present study.

In conclusion, PfHRP2-based RDTs are not recommended to be used in Colombian Amazonas because of the high percentage of isolates (38.5%) with deletion of both *pfhrp2* and *pfhrp3* genes. Monitoring the potential expansion of *pfhrp2*-negative parasites should continue in Colombia and RDTs that target pLDH and other target antigen should be used as an alternative. It is also possible that *pfhrp2*-negative parasites are present in other regions of the Americas where they have so far not been reported. This could be due to human migration from Amazonas and, more recently, due to extensive mining occurring in the greater Amazon region. Therefore, epidemiological studies and systematic molecular surveillance for *pfhrp2*-negative parasites in the greater Amazon region should be implemented. In addition, we highlight the importance of the genotyping of *P*. *falciparum* parasite in the Americas to assist the study of imported malaria cases and malaria outbreak investigations.
